# Feasibility, utility, usability and acceptance of a multimodal telemonitoring for COVID-19 patients in general practitioners practices in Germany: a mixed methods study with patients

**DOI:** 10.1186/s12913-025-13455-5

**Published:** 2025-09-18

**Authors:** Zoe S. Oftring, Kim Deutsch, Svea Holtz, Susanne M. Köhler, Peter Jan Chabiera, Nurlan Dauletbaev, Lukas Niekrenz, Beate Sigrid Müller, Sebastian Kuhn

**Affiliations:** 1 Institute for Digital Medicine, Philipp’s University Marburg and University Clinic Giessen & Marburg, Baldingerstrasse 1, 35043 Marburg, Germany; 2 Department of Pediatrics, University Clinic Giessen & Marburg, Philipp’s University Marburg, Marburg, Germany; 3 Institute of Educational Science, Johannes Gutenberg University, Mainz, Germany; 4 Institute of General Practice, University Clinic Frankfurt, Goethe University, Frankfurt am Main, Germany; 5 Institute of Legal Medicine, University Clinic Frankfurt, Goethe University, Frankfurt am Main, Germany; 6 Department of Internal, Respiratory and Critical Care Medicine, Philipps University of Marburg, (Member of the German Center for Lung Research (DZL)), Marburg, Germany; 7 Department of Pediatrics, Faculty of Medicine and Health Sciences, McGill University, Montreal, QC Canada; 8 Department of Pneumology and Intensive Care Medicine, University Hospital Aachen, RWTH Aachen University, Aachen, Germany; 9 Institute of General Practice, University of Cologne, Faculty of Medicine and University Hospital Cologne, Cologne, Germany

**Keywords:** Remote monitoring, Digital health application, Telemonitoring, COVID-19, General practitioners, Healthcare providers, Qualitative interviews, Digital medicine, Wearables, Pandemic preparedness

## Abstract

**Background:**

During the COVID-19 pandemic, infected outpatients were at risk of declining at home without themselves and their general practitioner (GP) noticing, above all due to silent hypoxemia. To support patients in quarantine, telemonitoring solutions were developed for primary care in several countries. However, evidence on patient perceptions of COVID-19 telemonitoring in primary care settings remains limited. This prospective study evaluates COVID-19 outpatients’ experiences with and perception of the usability, utility and acceptance of an app-based telemonitoring in Germany, identifying key conditions for its successful implementation.

**Methods:**

To support home-isolated COVID-19 patients remotely, eight GP practices in Germany implemented a multimodal telemonitoring system. Telemonitoring consisted of an app with connected sensors to remotely measure vital signs and symptoms, with data transmitted to a GP telemedicine platform. Between January to December 2021, 34 COVID-19 outpatients participated in telemonitoring. Telemonitoring duration was 28 days for acute infection or up to 12 weeks for prolonged/post-acute symptoms. Afterwards, patients participated in a mixed-methods evaluation about their experiences consisting of semi-structured telephone interviews and an in-house questionnaire. Interviews were analyzed using qualitative content analysis, questionnaires were analyzed descriptively.

**Results:**

All patients (34/34) completed the study (female = 22/34, 65%; median_age_=50.5 years; range_age_=19–74, comorbidities present = 13/34). Patients generally viewed telemonitoring as feasible and beneficial, with high acceptance rates and a perception of the system as valuable and reassuring support during illness. Participants, even those with limited prior experience in recording health data, successfully managed the monitoring process. Key insights included patient expectations regarding GP data access, underscoring the importance of integrating patient perspectives into the design process of future telemonitoring solutions. Connectivity issues with sensors occasionally disrupted data collection. Generally, the results emphasize the importance of comprehensive onboarding and support structures to optimize telemonitoring effectiveness.

**Conclusions:**

This study demonstrates that app-based telemonitoring in primary care is a feasible, well-accepted intervention for COVID-19 outpatients, with patients perceiving it as valuable, supportive and reassuring. Findings emphasize the critical role of patient-centered design and strong support structures for successful telemonitoring integration into primary care. Lastly, these findings underscore the value of telemonitoring in pandemic preparedness, ensuring timely detection of patient deterioration and strengthening primary care resilience.

**Trial registration:**

The study was registered with the German Clinical Trials Register (DRKS00024604). The study was approved by the Ethics Committee of Goethe University Frankfurt (No. 20-1023, 18.01.2021), and written informed consent was obtained from all participants.

**Supplementary Information:**

The online version contains supplementary material available at 10.1186/s12913-025-13455-5.

## Background

When the COVID-19 pandemic hit the global community in 2020, healthcare systems worldwide were ill- or unprepared for a crisis of this extent. Especially under strain were general practitioners (GPs) as representatives of the primary care sector and first point of contact for COVID-19 patients. Knowledge about the disease was only emerging, vaccinations or treatment options did not yet exist and both personnel (GPs, hospital doctors, nurses, paramedics, other healthcare professionals) and organizational resources (personal protective equipment, PPE; testing kits, GP appointments, hospital beds for referral) were scarce. Rapidly after the pandemic onset, silent hypoxemia with blood oxygen saturation levels (SpO2) < 93% emerged as an indicator for symptom decline [1]. But without means to monitor patients in quarantine, outpatients were at risk of declining at home without themselves and their GP noticing.

In the majority of COVID-19 patients (81–90%), the course of the disease is mild [2, 3]. These patients are mainly treated symptomatically in an outpatient setting without the need for hospitalization. However, severe cases can lead to pneumonia, acute respiratory distress syndrome, hyperinflammation, thromboembolic events, sepsis/organ failure or death. Preexisting conditions such as hypertension, diabetes or COPD significantly increase the risk for severe COVID-19 disease [4]. Additionally, the discovery of post COVID syndrome and the associated complex patient needs put further strain on GPs. Solutions to monitor and timely identify the deterioration of patients at home amidst limited staff resources, limited possibilities for home visits and lockdown restrictions were needed.

Telemonitoring solutions for chronic diseases such as diabetes or asthma already existed. But during the pandemic, health care providers worldwide, amongst them GPs, began using telemonitoring also in the context of infectious diseases to manage the treatment of highly contagious patients. This helped reducing the risk of transmission and improving GPs’ ability to assess patients’ health status, especially that of their most vulnerable patients [5–7] and also to maintain care for other patients during lockdown [8]. Wearable sensors further promoted health status monitoring and recommendations for suitable sensors emerged [9]. Several telemonitoring solutions for COVID-19 patients have been described. The complexity of these approaches varied and ranged from iterative symptom assessment via paper and phone/video consultation [7, 10], to patients measuring vital parameters at home and reporting results to healthcare providers via phone/video consultation [1, 11–15]. More advanced strategies involved mobile apps and web platforms for symptom monitoring [16–20], sometimes in conjunction with digital devices for measuring vital parameters [21–26]. The most commonly used devices were pulse oximeters, along with thermometers and blood pressure monitors. Most devices required patients to enter their readings manually, with only one approach using Bluetooth-connected devices for automatic data transfer [22].

As described, telemonitoring for COVID-19 patients contains several interacting components and stakeholders, and can thus be characterized as *complex intervention* [27]. The implementation, effectiveness and evaluation of such interventions needs to consider the bi- and multidirectional relationship of the individual components and participants, as well as the relevance of user-centered design [28]. Previous findings have indicated that the implementation of telemonitoring should involve patients at an early stage to gain knowledge about their views at various points in the implementation process [29–31]. However, most studies have focused mainly on either collecting health data, or the perspective of specific groups of professionals [32–34]. While there is increasing evidence on patient experiences with telemonitoring for acute COVID-19 in hospital-associated settings [12–14, 17, 23, 24, 35–37], studies on GP-led telemonitoring remain scarce [15, 38]. To the best of our knowledge, no research has yet explored patient experiences with a complex, multimodal telemonitoring system that integrates symptom monitoring, connected sensors for vital parameters, a patient app, and a medical platform. Thus, although the patient’s experience is crucial to adherence and the successful use of telemonitoring, little is known about expectations, experience, and the perceived usability of such a complex intervention for the management of infectious diseases from the patient’s perspective in the primary care sector. Understanding patient experiences and expectations is essential not only for ensuring adherence and usability but also for establishing telemonitoring as a reliable and effective tool within broader pandemic preparedness frameworks.

As part of the prospective telemonitoring study COVID-19@home [5], we addressed this research gap. The aim of this feasibility study was to design, implement, and evaluate a telemonitoring treatment concept for COVID-19 patients and to identify factors that support the successful implementation and future integration of such a multimodal telemonitoring into patient care. This paper presents the results of a qualitative evaluation of the experience of acute and post-acute COVID-19 patients in Germany with this telemonitoring in conjunction with a quantitative survey, both focusing on feasibility, usability, utility and technological acceptance.

## Methods

### Study design and setting

The prospective mixed-methods study “COVID-19@home” was conducted from January to December 2021. Patients were recruited in collaboration with eight family practices. All practices were located in Frankfurt/Main, Germany. Inclusion criteria for patients were age ≥ 18 years, a positive polymerase chain reaction (PCR) test for SARS-CoV-2, sufficient German language skills, and access to a smartphone.

The duration of the telemonitoring was 28 days. If COVID symptoms persisted, patients could extend the telemonitoring for a total of 12 weeks. We also recruited patients with a history of PCR-confirmed COVID-19 for the 12-week study arm if they presented to the GPs practice with post-acute COVID symptoms after their acute infection phase. In the following, we will thus refer to the three study groups as (i) *acute (aCoV)*, (ii) *persistent (persCoV)* and (iii) *post-acute COVID-19* (*postCoV*) track. We avoid using the term “post or long COVID” due to two reasons. Firstly, at the time of the study, evidence on this condition was still emerging, and no official diagnostic definition had been established. Secondly, in hindsight it is not possible to confirm whether the study participants met the current WHO criteria for post COVID syndrome, defined as “the continuation or development of new symptoms three months after the initial SARS-CoV-2 infection, with symptoms lasting at least two months without another explanation” [39].

Information on the study was provided to patients while they were being tested for SARS-CoV-2 in their GP practice or presented to their GP due to prolonged COVID symptoms. When patients tested positive or presented with prolonged symptoms, and were interested in study participation, the study team asked them for their informed consent. The GPs identified patients for study enrolment based on the inclusion criteria mentioned above. A demographic and clinical description of the total study population has previously been published in a preprint [5]. The results of the qualitative evaluation of the GP perspective on this telemonitoring approach have been published separately [40].

### Telemonitoring setup and digital data collection

The telemonitoring consisted of the patient app SaniQ (Company Qurasoft GmbH, Koblenz, Germany), digital sensors and an online medical telemonitoring platform (SaniQ Praxis). The app is CE-classified as medical device (medical device directive I, MDD I) and is app-store listed (iOS, Android). It incorporates several functionalities: the connection of digital sensors for remote monitoring of relevant vital parameters, Patient-Reported Outcome Measures (PROMs)/questionnaires, (a)synchronous communication with GPs via chat, file sharing (images, medical reports), and structured data export. All data between patient and physician is exchanged through a General Data Protection Regulation (GDPR)-compliant interface.

**Fig. 1:**
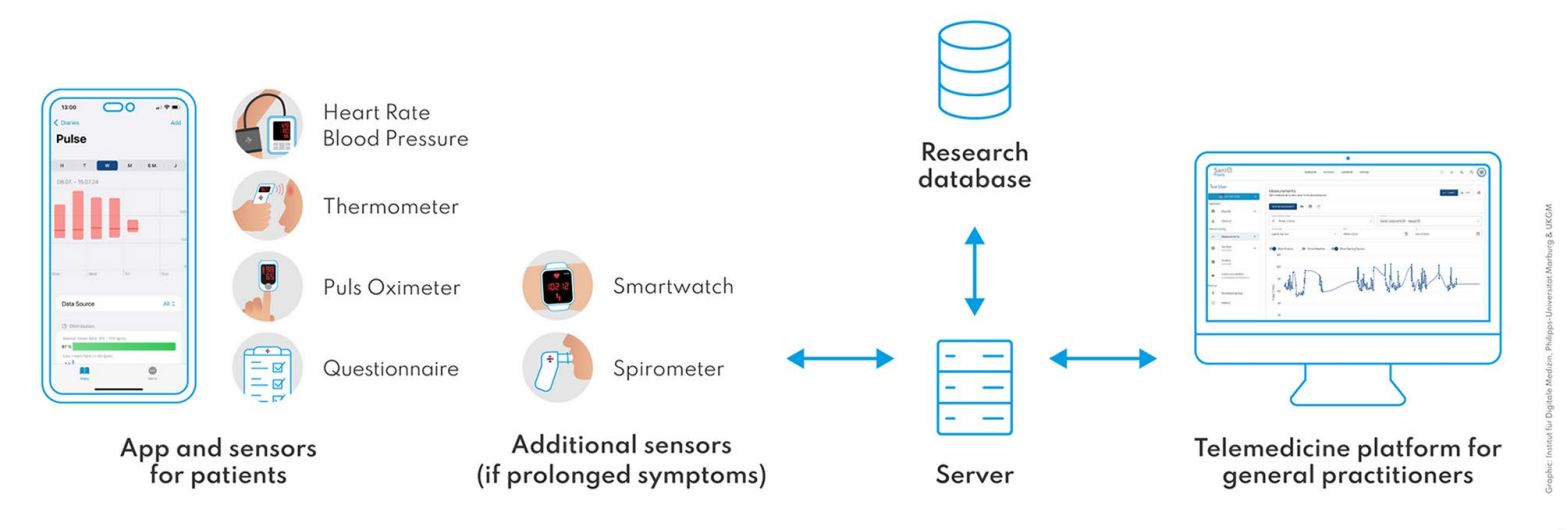
Schematic illustration of the telemonitoring structure with sensors (acute COVID-19 infection and prolonged symptoms)

The study team instructed the patients in the use of smartphone-based remote monitoring and provided them with download instructions, a registration code for the app and a leaflet containing information on setting up the application. By pairing the patient’s app with the physician’s telemonitoring platform GPs could remotely monitor the patients’ vital signs, symptoms and questionnaire answers. To measure vital parameters, each patient was provided with a sensor kit containing a pulse oximeter (Beurer medical PO 60), a blood pressure meter (Aponorm Basis Plus Bluetooth) and a non-contact fever thermometer (Beurer medical FT 95). Patients with persistent or post-acute COVID symptoms who entered the 12-week telemonitoring received additional sensors for this phase: an activity sensor wristband (Beurer active, AS 99) to measure heart rate and activity (daily steps), and a spirometer (MIR smart one) to measure the respiratory parameters peak expiratory flow (PEF) and forced expiratory volume (FEV1) (Fig. 1; and exemplary screenshots of telemonitoring workflow in additional file 1). The equipment was free of charge for patients. Patients measured themselves at least once daily with these sensors which were connected to the app and automatically transferred measurements via Bluetooth to the app and the physician’s telemonitoring platform. In case of technical problems, patients could enter measurements manually.

Beside the monitoring of vital parameters, symptoms were assessed using two questionnaires which were deployed through the app [5]. Upon enrollment, patients completed a customized medical history questionnaire evaluating their current COVID-19 symptoms and risk profile (relevant pre-existing medical conditions, current medication). This served as baseline assessment. It entailed 35 questions with yes/no response options, of which 14 evaluated baseline COVID-19 symptoms (see additional file 2). The instrument was developed by our study team based on the most recent scientific evidence about COVID-19 symptoms at the time of development [41–48]. Due to the rapid evolution of the pandemic and the urgent need for ambulatory studies, the first patients were enrolled while the questionnaire was still in development. Due to quarantine restrictions, all questionnaires were conducted via phone with the patients by a member of our study team who was also a medical doctor (SH) to ensure high medical standards. If patient statements were inconsistent regarding their stated pre-existing condition, it would prompt further inquiry by the doctor. For the initial 22 patients, the questionnaires were completed retrospectively. As new insights about COVID-19 symptoms emerged (e.g., skin rashes), additional symptom questions were added iteratively.

During the telemonitoring period, patients documented the progress of their symptoms daily in the app. At the beginning of the study (patient No. 1–16), patients tracked symptoms in free text in the in-app symptom diary, again due to a questionnaire being under development. Based on these free text answers and together with current guidelines of the participating pneumology departments and the German Society for General and Family Medicine (DEGAM) as well as validated questionnaires on fatigue and shortness of breath [5, 49–51], a daily symptom questionnaire was developed containing 7 questions (see additional file 3). It was implemented as daily in-app questionnaire for all consecutive patients.

Patients used the telemonitoring for at least 28 consecutive days, or 12 weeks in the prolonged COVID symptoms study arm.

GPs independently determined how frequently to monitor each patient’s data, based on the individual’s risk profile and disease progression, with a survey among participating GPs showing that patients’ data was at least checked daily [5, 40]. Thresholds for vital signs were set according to the standard operating procedures of participating pulmonology departments and the COVID-19 treatment guidelines available at the time [5, 52]. These predefined limits could be adjusted by GPs to better match the specific needs of their patients. If a patient’s vital signs exceeded or fell below these thresholds, the telemonitoring platform automatically sent an email alert to the responsible GP, who could then review the patient’s data and react accordingly [5]. A comprehensive description of the telemonitoring concept is available in the publication of Holtz et al. [5].

Support structures were in place for both GPs and patients. These were provided by the study team and the telemonitoring developer company to assist with any study-related questions or technical issues. Support was available by phone or via dedicated support section within the app during the day on weekdays. The study team advised patients with medical questions to contact their GPs and to call the national emergency hotline in case of a medical emergency.

### Mixed-methods evaluation

To gain holistic insights into the patient experience, the evaluation followed a mixed methods design consisting of qualitative interviews with the patients and a quantitative, inhouse questionnaire about the COVID-19@home telemonitoring experience.

#### Qualitative evaluation: semi-structured interviews

After the study period, all patients were invited to participate in a semi-structured telephone interview. Interviews were conducted to obtain in-depth feedback on the patients’ experiences with telemonitoring. For this, our research group developed an interview guide based on the modified Technology Acceptance Model (TAM) according to Gagnon et al. [53] and several interview studies in the realm of digital sensors and telemonitoring [54–56]. The modified TAM model evaluates technology acceptance by breaking it down into questions on *technological* (perceived usefulness, perceived usability, habits), *individual* (attitude, compatibility), and *organizational* (support, routine) issues. We chose Gagnon et al.‘s modified TAM, although it was originally developed for healthcare professionals, as its core constructs (e.g., perceived usefulness, ease of use) are equally applicable to patients in telemonitoring contexts, as they actively engage with the technology.

We also focused on collecting patient suggestions on potential improvements in order to derive specific recommendations for telemonitoring implementations. The final interview guide (see additional file 4) consisted of five domains (experience with telemonitoring (TAM), study setting, reflection, suggestions, conclusion) and contained questions on engagement of patients in self-measurement, perceived usefulness, ease of use, intention to use, compliance, attitudes towards technology, motivation, expectations, ability to deal with critical vital signs, concerns, obstacles, opportunities, and suggestions. An interdisciplinary group of researchers reviewed and revised the final interview guide before the patient interviews. All interviews were conducted by phone by a researcher with training in qualitative interviews, recorded and transcribed verbatim. The interview survey continued until data saturation was reached, defined as the point at which no new subcategories, themes, or insights emerged. The interview content was iteratively discussed within our research team to assess whether it sufficiently captured the multifaceted patient perspective and complexity of our research focus and to determine when saturation had been achieved.

#### Quantitative evaluation: in-house questionnaire

Additional to the interviews, patients evaluated their experience and satisfaction with telemonitoring after study completion using an in-house developed questionnaire. The questionnaire was developed by our study team based on recent instruments for the assessment of patient satisfaction and usability related to telemedicine or remote monitoring technologies [31, 57–59]. It consisted of 11 items in the four domains *usage of patient app and medical devices* (in the following ‘telemonitoring’), *patient satisfaction with the telemonitoring*, *previous experiences with telemedicine*, and their *general health during the acute COVID-19 infection.* The items were developed to mirror relevant parts of the semi-structured interview guide and thus allow triangulation. The questionnaire included both yes/no and 4-point Likert-Scale response options. A 4-point Likert scale was chosen to encourage clear responses regarding user-experience, while also ensuring robust data interpretation despite a relatively small sample size. The questionnaire was adapted for the digital format, allowing patients to complete it in the patient app (see additional file 5).

### Data analysis

Qualitative interview data were analyzed with an inductive-deductive content analysis approach according to Mayring [60] and Kuckartz [61–63]. The qualitative research group consisted of five researchers with training in qualitative methods (KD, ZSO, LN, SH, PJC). MAXQDA© coding software (version Analytics Pro 2020, VERBI Software GmbH, Berlin, Germany) was used for the analysis.

**Fig. 2:**
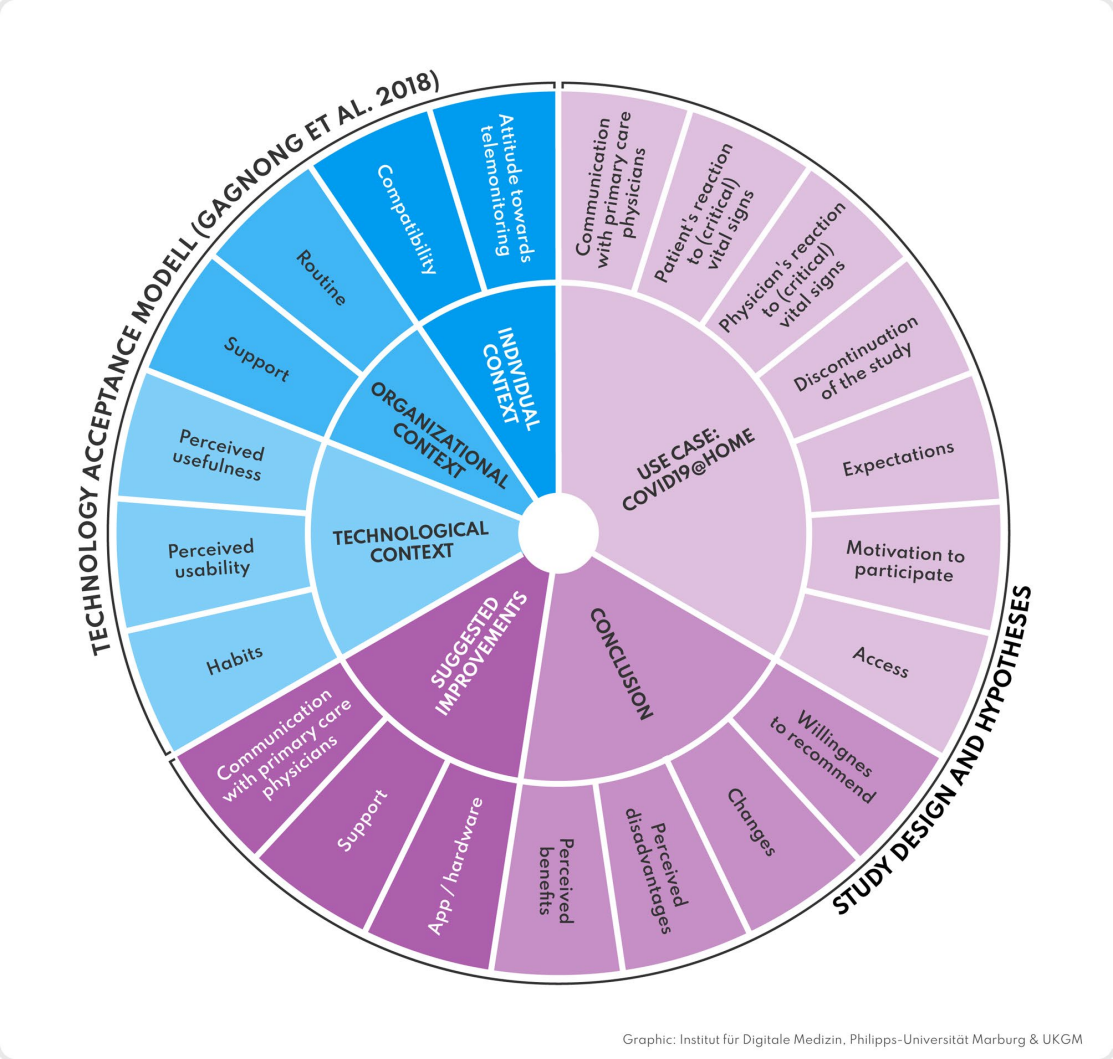
Final category system with main categories, subcategories and the theoretical coding framework. *Legend*. Inner circle = main categories; outer circle = subcategories; outer edge = theoretical framework. *Note*. The size of the category segments is purely descriptive and does not show a proportional distribution of results

Before the coding process, we developed a category system based on the interview guide and the TAM framework [53]. The first three interviews were coded according to the deductive category system by three researchers from our group. Subsequently, the findings were discussed and the category system slightly adjusted inductively (Fig. 2).

Thereafter, the remaining interview transcripts were individually coded by the researchers. We discussed findings and data saturation in weekly meetings with all researchers. After completion of the individual interview coding process, the individual MAXQDA project files were merged into a final project file. This master file formed the basis of the summary grid, in which all categories were divided up among our research group and summarized in terms of content [63]. The summary grid was first used category-by-category and then as an analysis tool for the subsequent interpretation across categories. Interim results and further steps were regularly discussed by the researchers and project supervisors. The report of the qualitative analysis process in this paper follows the COREQ quality standards [64].

Quantitative data (symptoms, in-house questionnaire) were analyzed descriptively using MS Excel 2016 (Microsoft, Redmond, WA, USA).

### Ethics approval and study registration

The study was approved by the ethics committee of the Goethe University Frankfurt (Ref. No. 20-1023). Written consent was obtained from all participating patients and general practitioners. The study was registered with the German Clinical Trials Register (DRKS00024604). COVID-19@home is part of “egePan Unimed” [65] and was funded by the Federal Ministry of Education and Research (BMBF) as part of the Network of University Medicine (NUM) (funding reference: 01KX2021).

## Results

### Participants

When we interviewed patients after they finished the telemonitoring period, qualitative data saturation was reached after 2/3 (67%) of the 51 patients participating in the COVID-19@home study. This paper presents the results of the evaluation of these 34 interviews. The population consisted of 22 women (65%) and 12 men (35%), patients were between 19 and 74 years old, with a median age of 50.5 years. Regarding monitoring duration, 27/34 patients (79%) were monitored for 28 days during their acute COVID-19 infection. Eight of them extended their participation due to prolonged symptoms to the 12-week period, and seven entered the study only due to persistent or newly developed symptoms although their acute infection was over.

The baseline symptom and health status assessment questionnaire that patients filled in upon enrolment revealed that several patients had risk factors for a potentially severe course of COVID-19 such as obesity (12%), or a critical pre-existing condition such as diabetes mellitus (7%), pulmonary disease (13%) or cardiovascular disease (7%). Preexisting conditions were present in 38% of patients. Eight patients (24%) reported having one pre-existing condition, four had two conditions (12%), and one (3%) had three relevant pre-existing conditions upon inclusion in the study. No patient was pregnant at the time of the study (Table 1). Study participants reported a median of five COVID-19 associated symptoms upon enrolment, with the three most prevalent symptoms being lethargy/exhaustion (84%), headache/dizziness (73%) and muscle/joint pain (67%) (Table 1).

**Table 1:** COVID-19 disease – patient baseline symptom and health status assessment (*n* = 33, missing = 1 due to questionnaire non-completion)

COVID-19 related symptoms at disease onset – Questionnaire Item^1^	Symptom prevalent		Valid^2^/Missing
	*n*	%	*n*
Lethargy/exhaustion	26	84	31/02
Headache/dizziness	11	73	15/18
Pain in muscles/joints	20	67	30/03
Cough	21	64	33/-
Difficulty concentrating	9	60	15/17
Smell/taste impairment	12	38	32/01
Fever/chills	11	33	33/-
Sore throat	11	34	32/01
Gastro-intestinal complaints	8	25	32/01
Rhinitis	13	42	31/02
Shortness of breath/dyspnoea	9	28	32/01
Chest pain	5	16	32/01
Skin rash	4	14	28/05
**Pre-existing conditions**	**Condition prevalent**		**Valid/Missing**
Diabetes mellitus	2	7	31/02
Cardiovascular disease (e.g. hypertension, cardiac disease, status post cardiac arrest)	2	7	31/02
Pulmonary disease (e.g. asthma, COPD, cystic fibrosis, pulmonary fibrosis, sarcoidosis)	4	13	31/02
Chronic liver disease	1	3	31/02
Oncological disease	1	3	31/02
Immunodeficiency (due to disease or medication)	0	0	31/02
Thyroid disease	8	26	31/02
Haematological disease (e.g. anaemia)	1	3	31/02
Neurological disease (e.g. dementia, status post stroke)	0	0	31/02
Pregnancy	0	0	33/-
Smoking	1	3	30/03

^1^ See additional file 2

^2^ The differences in the valid total numbers are due to iterative updates of the questionnaire based on the medical histories of the patients in the process of the study

*Note* All percentages are rounded

### Qualitative evaluation

The mean duration of the 34 interviews was 16.8 min (range 6.5–34.0 min). This narrative report presents patients’ perspectives on the telemonitoring, focussing on the domains (i) perceived usefulness and ease of use, (ii) perceived benefits, (iii) perceived disadvantages and (iv) suggestions for improvement. These domains were created to align with the research question. The findings from each main category informed the results across all four domains. Anchor examples from the interviews support the report. The original quotes were in German and translated to English for this paper, minor changes were made for better readability. Displayed in parentheses is the patient ID which consist of the individual patient number and an indicator of the monitoring path (participated in the aCoV = acute COVID-19 track, *n* = 19; persCoV = the persistent COVID-19 symptoms track, *n* = 8; or postCoV = the post-acute COVID-19 symptoms track, *n* = 7).

#### Patients’ experiences of using telemonitoring: perceived usefulness and ease of use

For patients, the perceived usefulness of telemonitoring lies in the objectification of symptoms through the use of sensors and continuous measurements to track their vital signs.

*I just kept having a look to see how things had progressed in the last month. You got a pretty good overview of what your values had been like before. And that was quite interesting for me - what had changed*,* actually in a positive sense. (020_aCoV)*

Measuring vital signs fostered a sense of self-control and increased patient awareness of their disease progression. This contributed to a feeling of safety regarding their condition, while the app’s documentation feature encouraged patients to monitoring themselves regularly. The possibility to combine various app features, such as recording vital signs and documenting medications simultaneously, was also found to be useful. Overall, operating the devices and using the app were described as simple and intuitive. However, patients occasionally experienced difficulties with pairing the devices to the smartphone and with incomplete or failed data transfers. The most commonly cited problems were related to transmitting data from the Pulse Oximeter and Activity Wristband. Two interviewees reported that a poor internet connection and the distance between sensor and end device negatively impacted the transfer. Nevertheless, participants were solution-oriented and either manually entered results into the app or repeated the measurements in case of transmission problems.

*Well*,* I let blood pressure measurements go through Bluetooth all the time*,* that worked very well. But I entered body temperature and oxygen saturation manually. (018_aCoV)*

Experiences with the initial setup of the devices and the app varied. Most patients reported no difficulties from the start. In some cases, misunderstandings caused difficulties in the beginning but could be quickly resolved with assistance from the support team, leading to smooth app usage.

#### Patients’ experiences with telemonitoring: perceived benefits

Patients described a range of perceived benefits, which can be grouped into three subcategories: treatment decisions, improved understanding of disease progression and lifeline to GPs.

Patients reported that their self-collected data prompted them to make treatment decisions, either independently or in consultation with their GP.

*It was very reassuring for me that I could monitor myself. And to be honest*,* I didn’t have to go to hospital. I can say that I was able to manage everything a bit myself. I would probably have said to myself: ‘No*,* I’m going to go this far and no further*,* now I’m really going*,* or I’ll admit myself to hospital’*,* I would probably have decided that for myself. But all these measurements actually contributed to the fact that I was actually able to decide everything for myself or could have decided with the doctor. (013_persCoV)*

*So*,* I still haven’t seen a lung specialist*,* but I was given medication based on the value I measured at home [with the spirometer]. That wouldn’t have happened without these devices. (019_persCoV)*

Patients felt empowered to take an active role in managing their illness and were willing to assume greater responsibility, provided certain conditions were met: they emphasized the importance of reference ranges for accurately interpreting their vital signs and believed that the reliability of these measurements depended on consistent data collection over time. However, in some cases, patients preferred to defer treatment decisions, viewing the interpretation of data and any necessary medical interventions as the sole responsibility of their GP.

*As a layman*,* I couldn’t judge whether the values were good or bad or elevated. I entered the values and just thought*,* well*,* if there was anything alarming*,* my doctor would get in touch with me. (028_aCoV)*

A further benefit was the improved understanding of disease progression through objective digital vital signs. Patients reported that the self-collected data gave them a sense of control, which was reassuring as stated in almost all interviews, particularly in light of media reports and the known risks of COVID-19 infection. The reassuring effect of stable blood oxygenation levels proved by oximeter measurements was repeatedly mentioned. Overall, the interviews emphasized reduced anxiety and increased safety through telemonitoring.

*Well*,* that you [get something]*,* yes*,* like a first-aid kit. […] you are put in quarantine and then you are left at home with your illness. Of course*,* you can go to the doctor if you feel really bad. But then you’re back in this special room. You have to be because of the rules on hygiene. But you just don’t feel you’re in good hands at that moment. And that has nothing to do with the practice or the doctor*,* only with the rules on hygiene. And if you could have the feeling that you were able to take your own measurements and then be in contact with the doctor or with all the medical staff*,* then I think that could help reduce a lot of people’s anxiety. (027_persCoV)*

Visualization of health improvements through retrospective measurement curves in the app tended to enhance patients’ subjective well-being. Some patients even described its impact beyond the acute COVID-19 infection: The information about their vital signs was motivating and encouraged them to improve their overall health and lifestyle, such as increasing their daily steps. Other patients resorted to their health data when seeing other specialists to complement their medical history. This indicates a heightened awareness and understanding of health, or health literacy. In this context, recording objective health data can serve as a foundation for adopting a more proactive and sensitive approach to personal health.

The feedback also underscored the perceived benefits of involving their GP in the telemonitoring. Patients appreciated that their doctor’s office could access collected data and were generally receptive to sharing health information for their care. Benefits included fast and easy data access, simplified communication with the GP via the app, the regular data collection irrespective of location and time, the reduction of in-person doctor visits (especially during a time that patients felt unwell and did not want to leave the house) and the perceived modern care approach.

*I think it’s very*,* very modern and absolutely forward-thinking and I think it’s good that something like this exists; that clever minds get together and work something out and put something together that simply helps normal people*,* like me*,* and maybe also helps the community to get through all this crap. (ID 18_aCoV)*

Patients also noted the added benefit of telemonitoring to their overall health due to the continuous monitoring of relevant parameters to their GP. They emphasized that the telemonitoring provided GPs with better information which could unmask other healthcare problems such as detecting high blood pressure and thus support GPs in providing preventive care. Furthermore, data collected at home in patients’ usual environment was considered more reflective of their condition and thus “closer to reality”.

*So*,* overall*,* I think it’s good that you can take measurements at home in such a protected environment. Well*,* because he [GP] also said to me that if you measure your blood pressure in the practice*,* it’s usually always too high or higher. Because you automatically get excited when you’re sitting opposite the doctor. […] this example of body temperature*,* you can’t falsify it - but blood pressure is something where it can be incredibly helpful if you can record the value for a week using an app and the existing blood pressure monitor. And I believe that a doctor can do much better with the data than with a random sample*,* which is then additionally falsified because someone is sitting there in the treatment room with a certain amount of excitement. So*,* I think that’s something that really speaks in favor of telemonitoring. (040_postCoV)*

#### Patients’ experiences with telemonitoring: perceived disadvantages

Beside all the benefits, patients also perceived some disadvantages. The majority of these related to technical aspects, and significantly fewer to personal and organizational/personnel aspects.

Technical aspects concerned data transfer and synchronization, measurements inaccuracies, and data protection. The majority of patients praised the measuring process with the blood pressure machine, thermometer and spirometer to be user-friendly and smooth, but occasional issues arose around the oximeter and the activity tracker. Here, inaccurate measurements or incomplete data transfer to the app caused frustration or insecurities and raised doubts about the devices’ sensitivity.

In relation to digital technologies data protection in general was raised as concern in some interviews, although addressed during the onboarding process and in the form of consent document.

*Yes*,* the disadvantage is of course data protection. You can never be 100% sure with any app*,* whatever they may say*,* what is going to happen with the data*,* right? (010_aCoV)*

Patients described privacy as a necessary condition of data collection, viewed the app’s server location in Germany as trustworthy and expressed their motivation to contribute to the research on the new SARS-CoV-2 virus by providing their data in a study.

Lastly, when patients reflected on the technology, some hypothesized that older or less technology-savvy people may experience problems with telemonitoring.

On a personal level, patients described the effect of technical issues. One case illustrated that these measurements also involved a strong psychological component in what was – back then – a seemingly unpredictable disease:

*I think it was the tenth day that I felt really bad. […] I was perhaps also*,* I don’t want to say panicking*,* but something in between worry and panic. Also because of the values. So*,* with this little measuring device [oximeter] in particular*,* I had looked at it several times. And I had always hoped—well*,* I didn’t want to go to hospital either*,* I have to say*,* I was somehow quite worried because it was also quite a high phase [of the pandemic] and you’d already heard that you don’t know which hospital you’ll end up in*,* God knows where. Always the hope that you would stay above 90 [percent blood saturation] with this device. (004_aCoV)*

Others expressed the implications of technical problems on their user behavior. In cases of connectivity problems, data collection was experienced as frustrating and technically demanding, occasionally necessitating support. Technical issues such as low battery capacity or the necessity to repeatedly enter results manually also decreased patients’ willingness to perform measurements.

Some respondents noted that setting up the equipment, familiarizing with the technology and taking measurements was particularly challenging during a time of acute illness and symptom burden.

*When you’re not feeling well and are not in the mood to do it*,* then it is hard work. And when the data don’t transfer properly the first-time time*,* or if there are other problems*,* then you get irritated. (005_aCoV)*

While most patients found it easy to integrate telemonitoring into their daily lives, estimating an effort of about 10 min per day, a few patients still found the measurement process rather time-consuming and the integration into their daily lives challenging.

Another perceived disadvantage related to data management by GPs. Patients were dissatisfied when their GP neglected to review their collected data. Furthermore, patients noted that the collected data conveyed an incomplete picture of their condition, as their vital signs might be stable, yet they would experience considerable discomfort and symptom burden. Some expressed a preference for brief follow-ups and reassurance from their GP, and some suggested an additional communication channel between doctor and patient for this – thereby revealing unawareness of the chat function included in the app albeit the thorough onboarding upon enrollment that all patients received.

#### Patients’ experiences with implementing telemonitoring: suggestions for improvement

After the analysis of perceived benefits and disadvantages and together with statements from the category “suggested improvements”, suggestions for future telemonitoring implementations were derived. These can be categories into three subcategories: (i) *app/hardware*, (ii) *study organization and support*, and (iii) *interaction with GP*.

(i) The interviews revealed that some participants were unaware of relevant app features as they suggested integrating a symptom diary allowing for free-text entries, e.g., to document uncommon COVID-19 symptoms, a messenger feature to communicate with their GP, and an option to export and subsequently print their data. All these were options offered by the app but seemingly not discovered by all participants. This shows the relevance of the onboarding process. Although all patients received thorough onboarding upon enrollment, the evaluation revealed that an improved and standardized onboarding process is needed. Additionally, participants of complex interventions should be equipped with more informative resources about the study and devices (e.g., user manual, leaflet, online video tutorial) that they can refer to if questions arise and that considers age-specific requirements (e.g., font size, hearing impairment).

Hardware-related problems resulted from differences between the Android and iOS operating systems. Subsequently, practitioners and technical support structures need to consider system-specific differences and especially inquire about the smartphone in use when giving support without being onsite.

(ii) Study-related suggestions focused on the timing of the daily symptom questionnaire notifications. According to patients’ recommendations, daily symptom questionnaires should be sent around midday or afternoon, as earlier scheduling made meaningful symptom reports difficult, or should alternatively cover the day before. Additionally, it was noted that the symptom questionnaire did not adequately capture post-acute COVID specific symptoms. At the time of the study, the knowledge about sequelae of COVID-19 was only emerging and specific PROMs did not exist. However, future telemonitoring concepts of severe infectious diseases should consider the potential for such consequences and screen patients accordingly.

Regarding technical support, patients recommended direct contact possibilities to the software company through a hotline, as this would avoid three-way communication.

(iii) Suggestions for improved interactions with GPs centered on the wish for more feedback on vital signs. Patients emphasized the need for selective rather than continuous, feedback, for example in cases where vital signs had significantly worsened. Additionally, patients were dissatisfied when GPs did not review their collected data before treatment decisions were made. Although these incidents were isolated and not a general negligence, it needs to be emphasized that the medical responsibility to check results and react accordingly equally applies to telemonitoring as it does to all medical interventions. Therefore, digital parameters need to be carefully curated. To facilitate this, alerts and notifications in the platform and via email should made use of. From a public health perspective, it is necessary that the GPs’ efforts related to telemonitoring does not become an additional burden but is compensated by insurance, only then will modern technologies like this be integrated on large-scale to optimize patient care.

### Quantitative evaluation

**Fig. 3:**
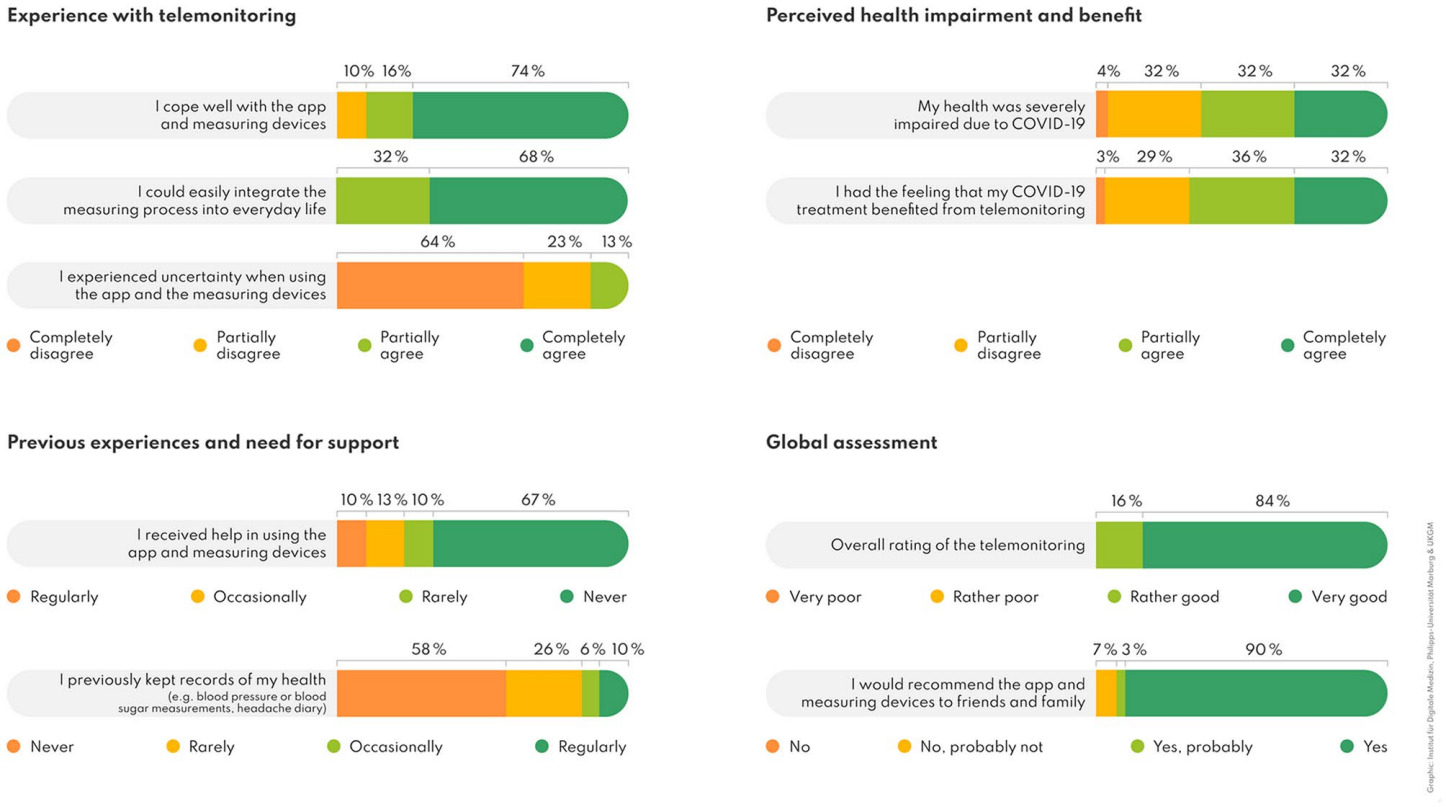
Patients’ (*n* = 31) experience with the telemonitoring and overall assessment based on the in-house questionnaire results. *Note* All percentages are rounded

In total, 31 of the 34 patients (91%) answered the in-house evaluation questionnaire. Regarding digital health literacy, most study participants (84%; 26/31) had “rarely” or “never” previously made records of their health data. Nevertheless, most of them “completely agreed” or “partially agreed” that they had managed to cope with the app and measuring devices (90%; 28/31), while 68% (21/31) “completely agreed” and 32% (10/31) “partially agreed” that they “could comfortably incorporate the measurements” into their daily lives. Most patients dealt with the app and devices without assistance (77%; 24/31) and the vast majority (87%; 27/31) “partially disagreed” or “disagreed” to have experienced uncertainty whilst using the app. Patients’ overall opinion of home monitoring was very positive with an overall assessment as “very good” by 84% (26/31) and as “rather good” by 16% (5/31) (see Fig. 3).

Following the mixed-methods approach, quantitative (*n* = 31) and qualitative (*n* = 34) findings were triangulated: Both surveys revealed a positive or very positive experience with the telemonitoring, while a minority of participants experience uncertainties or difficulties. Equally, participants predominantly used the telemonitoring autonomously but occasionally required support. The questionnaire survey provided a more quantified picture of the disease burden compared to the interviews with 64% agreeing to have experienced severe impairment due to COVID-19 and more than two-thirds (68%) agreed that the telemonitoring benefitted their illness. Both evaluations polled willingness to recommend. According to the questionnaire, 28/31 patients (90%) would definitely, and 1/31 (3%) probably recommend the app and measuring devices to family and friends, while 2/31 (7%) participants would probably not. The qualitative interview responses were divided into the four categories (i) recommendation without limitations, (ii) recommendation with limitations, (iii) indecisive, and (iv) no recommendation. Overall, 26/34 (76%) participants would recommend it without limitations and 8/34 (24%) with limitations. Connectivity and synchronization problems between sensors and the app were listed as limiting factor. No patient was indecisive or indicated that they would not recommend the telemonitoring.

In summary, the results of the quantitative evaluation regarding the willingness to recommend telemonitoring largely parallel the interview results and show a high acceptance of telemonitoring and its perception as a valuable support tool during and after an acute COVID-19 illness.

## Discussion

### Principal findings

The COVID-19 pandemic challenged healthcare systems to support infectious patients and timely detect deterioration despite quarantine restrictions and scarce resources. To better monitor COVID-19 patients, we implemented an app- and sensor-based telemonitoring. Our results demonstrated the feasibility both for acute and prolonged COVID-19 cases, suggesting its potential for general pandemic preparedness strategies. High acceptance rates and the perceived usefulness suggests strong patient satisfaction and interest in this concept. A comprehensive onboarding and support structures emerged as essential success factors. It proved beneficial to ground the sensor selection and questionnaire development in existing evidence and literature. Nevertheless, COVID-19’s novelty necessitated iterative, dynamic adjustments to new scientific findings. Sensors were predominantly perceived as user-friendly, however connectivity problems occasionally impacted data transfer and patient adherence. Human factors need to be considered and balance between sensor count, monitoring extent and effort is paramount. Our findings unveiled the psycho-emotional factors in telemonitoring. Patients felt empowered by contributing data to their doctors, reassured by normal readings, and distressed by critical ones, underscoring the importance of clear patient guidance, medical fallbacks (e.g., emergency number), and personal GP support during unpredictable illnesses. Patients found telemonitoring highly beneficial when data informed care and disappointing when data was overlooked. The GP perspective revealed that many GPs found telemonitoring useful, while others saw limited value due to the added time required, often outside consultation hours [40]. We hypothesized that GPs faced immense strain during the pandemic’s peak, supporting COVID-19 patients alongside regular acute and chronic cases. Lack of experience with telemedical workflows and reimbursement models may have hindered its full utilization. In any telemonitoring intervention, physicians need to treat telemonitoring data with the same responsibility as other medical results, and reimbursement models need to acknowledge additional expenses on medical side as existent for other conditions such as chronic heart failure [66–68].

### Comparison to prior work

Our results clearly indicate the feasibility, perceived benefit and patient satisfaction with telemonitoring as an additional component in the support of patients with an infectious, unpredictable and potentially fatal disease. These findings parallel existing research, demonstrating high acceptance levels for COVID-19 telemonitoring [12, 32, 34] and emphasizing its potential to timely detect deterioration, support treatment decisions [1, 13, 15, 24], monitor patients after hospitalization [22] and provide reassurance [36, 38].

Qualitative evaluation results underscore that telemonitoring’s effectiveness depends significantly on supportive structures. Previous studies also highlighted this [35] together with the collaboration between all stakeholders to foster universal implementation and optimize data flow [19, 69]. In our study, close cooperation between the study team, GPs and software developers was established and maintained throughout the study. Swift response to technical problems and questions significantly influenced patients’ attitudes and strengthened adherence. Under methodological aspects, the interview results highlight the importance of adopting qualitative methods in telemedicine feasibility studies to gain a detailed understanding of patients’ experiences. Given the anticipated rise in telemedicine, these insights are essential to guide effective implementation and practices. Although two similar multidimensional COVID-19 telemonitoring concepts (app, multiple sensors, medical platform) have been published [22, 70], no study has yet qualitatively analyzed the patient perspective in the GP sector. Silven et al. describe the pathway and implementation of a similar COVID-19 telemonitoring lead by clinicians but did not evaluate the patient perspective [22]. Cerdan de las Heras et al. conducted a mixed-methods pre-test study of a home monitoring concept with hospitalized in-patients [70]. However, the pilot and hospital setting limit the results’ transferability to real outpatient settings. Publications on the patient perspective in the GP setting include less complex telemonitoring concepts or narrower qualitative evaluation. Nevertheless, the results overlap with our findings, particularly with respect to an increase in patients’ perceived safety, utility, reassurance and the necessity for comprehensive onboarding [15, 36, 38].

### Implications

The COVID-19 pandemic demonstrated the need for rapid-deployment solutions to support large numbers of infectious patients. Healthcare leaders should consider national emergency telemonitoring plans, akin to Germany’s “Corona Warning App” [71]. Medical training must equip professionals with the skills, knowledge and competencies to implement and utilize these technologies effectively.

Healthcare innovations must uphold ethical, equality and equity standards to benefit the greatest patient number possible. While components were cost-free, participants required a smartphone and basic technical skills for the setup. Furthermore, patients with language barriers or sensory disabilities were unable to participate. Future telemonitoring should aim to minimize digital divide and enhance accessibility.

Lastly, interviewees statements during the pandemic peak indicated the necessity for support structures for post-COVID syndrome (PCS). Due to PCS’s novelty, our telemonitoring concept did not initially differentiate between conditions, but repetitive symptom assessment allowed us to capture changing patient needs. These insights contributed to developing a pediatric PCS telemonitoring pathway [72], with adult PCS surveillance currently under study [73, 74]. Further research is necessary to fully leverage the benefits of telemonitoring for PCS.

### Strengths and limitations

The most important strength of our study was the patient-centred approach to implementation complemented by in-depth interviews that captured the multifaceted reality of the patient experience. The high interview number resulted in data saturation, contributing to the study’s validity. While the relatively small sample size is a frequently cited limitation of qualitative research, according to Helfferich [75], an appropriate range lies between 6 and 120 participants. Within this framework, our sample of 34 patients can be considered adequate to support the validity of the findings. Medically, all tools except the smartwatch were certified medical devices and GDPR-compliant, attesting to the concept’s high standard. Smartwatch data was considered observational data and not used for direct medical judgment. Our study confirmed the user-friendliness of the telemonitoring and its feasibility shortly after diagnosis. Though adherence analysis would have benefited from data on daily parameter transmission, the inclusion of a diverse adult population, with patients of all age groups, greatly varying degrees of health data recording experience and varying degrees of pre-existing conditions, supports the generalizability of our results to the German primary care context. However, the relatively healthy cohort limits conclusions for multimorbid patients, and a deeper assessment of digital health literacy would have strengthened the study. There was a slight predominance of female patients and limitations in terms of inclusiveness, nevertheless good generalizability of the findings for the German primary care sector can be assumed. Conclusively, the telemonitoring is suitable for broad segments of the population, highlighting its potential to be a successful part of pandemic preparedness.

Beside these benefits, this study is not without limitations. First, telemonitoring was voluntary, potentially introducing selection bias and reducing generalizability as our sample could have been favorably disposed towards participation. Second, digital divide and accessibility limitations have already been discussed above. Third, under methodological aspects, we deliberately chose the Technology Acceptance Model due to its simplicity, strong empirical grounding, and suitability for the exploratory nature of our study. However, we acknowledge that the Unified Theory of Acceptance and Use of Technology (UTAUT) has been shown to offer greater explanatory power in some health technology adoption studies [76] and will include it in future studies. Additionally, the in-house questionnaire was developed by our study team to allow quantitative measuring of user-experience and perspective as well as triangulation. However, its lack of validation may have limited evidence. Direct interaction in telephone interviews may introduce social desirability bias. However, strong alignment between quantitative and qualitative results minimizes this concern. Lastly, the analysis excluded cost-effectiveness, limiting the results under economical perspectives. However, despite these limitations, our study constitutes the first exploration of patient experiences with a multidimensional telemonitoring concept for COVID-19 in Germany.

## Conclusion

This study demonstrates that app-based telemonitoring is feasible for patients with acute COVID-19 and prolonged COVID-19 symptoms in a primary care sector. Telemonitoring leads to an increase in patient safety as it enables physicians to timely detect deterioration through daily vital parameters and was highly accepted and perceived as beneficial and reassuring by patients. Our findings identified patient-centered design, comprehensive onboarding and strong support structures as critical facilitators of telemonitoring’s effectiveness.

Prospectively, this telemonitoring approach is transferable to other acute, infectious diseases and further research should address telemonitoring as part of a national emergency healthcare plan for pandemics. Lastly, this study encourages further research on telemonitoring solutions for post-acute and post-COVID syndrome patients.

## Supplementary Information

Below is the link to the electronic supplementary material.

Supplementary Material 1

Supplementary Material 2

Supplementary Material 3

Supplementary Material 4

Supplementary Material 5

## Data Availability

The generated and analyzed interview datasets are not publicly available due to the need to protect participants’ privacy and confidentiality.

## Abbreviations

aCoV: Acute COVID-19 symptoms
COVID-19: Coronavirus disease 2019
FEV1: Forced Expiratory Volume
GDPR: General Data Protection Regulation
GP: General Practitioner
MDD: Medical device directive
TAM: Technology Acceptance Modell
PCS: Post-COVID syndrome
PEF: Peak expiratory flow
persCoV: Persistent COVID-19 symptoms
postCoV: Post-acute COVID-19 symptoms
