# Supplementary material for: Feasibility, utility, usability and acceptance of a multimodal telemonitoring for COVID-19 patients in general practitioners practices in Germany: a mixed methods study with patients
Source: BMC Health Serv Res. 2025 Sep 18;25:1203. doi: 10.1186/s12913-025-13455-5 (PMC12447617; doi:10.1186/s12913-025-13455-5)
Supplement: Supplementary file 1 — Supplementary Material 1 [file 12913_2025_13455_MOESM1_ESM.pdf]

Additional file 1. Exemplary Screenshots of the telemonitoring functionalities.

SaniQ Patient App

a) Start Screen
b) Home Screen showing vital parameter diaries
c) Smartwatch Puls data measurements
d) Pulse oximeter measurements (SpO2)
e) Spirometry measurements (FEV)
f) File sharing

Online Physician Telemonitoring Platform

g) Start Screen – Login
h) Overview spirometry measurements (FEV)
i) Overview step count
j) Chat function

Note: For privacy reasons, screenshots **do not** depict real study patient data but demonstrational data. Time periods might therefore not align with the study period.

Image source: Screenshots SaniQ Qurasoft App; SaniQ Telemedicine online platform.

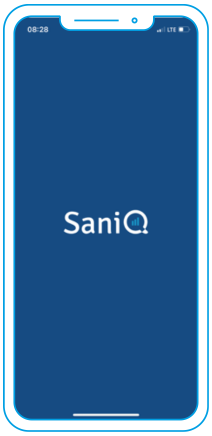

a)

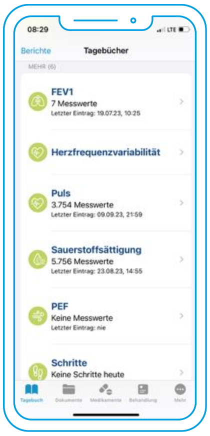

b)

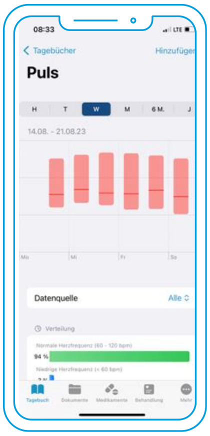

c)

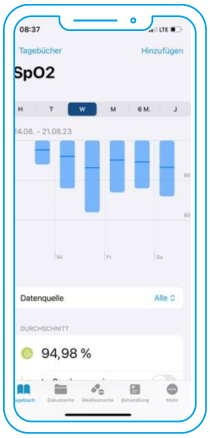

d)

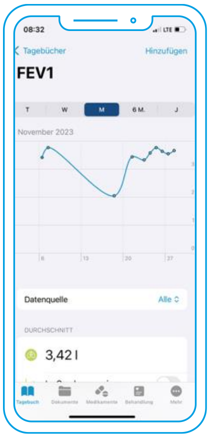

e)

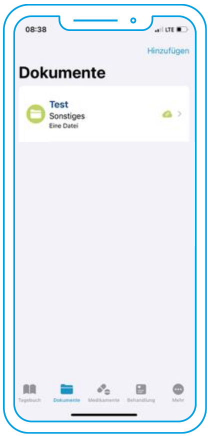

f)

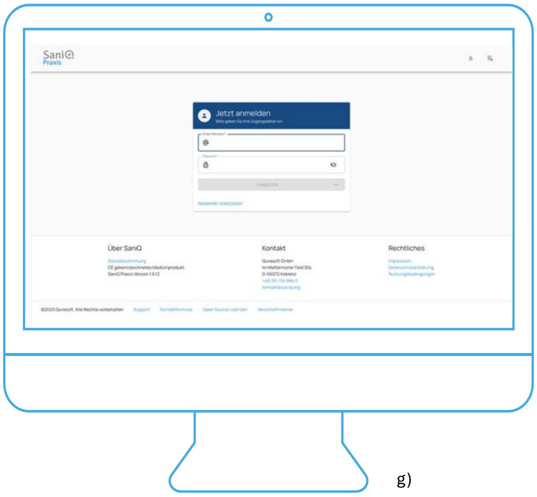

g)

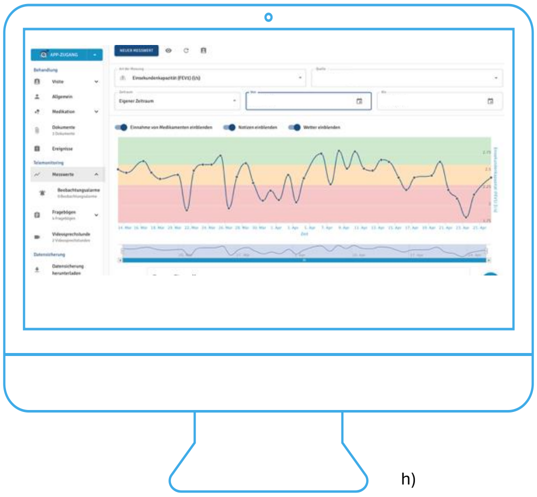

h)

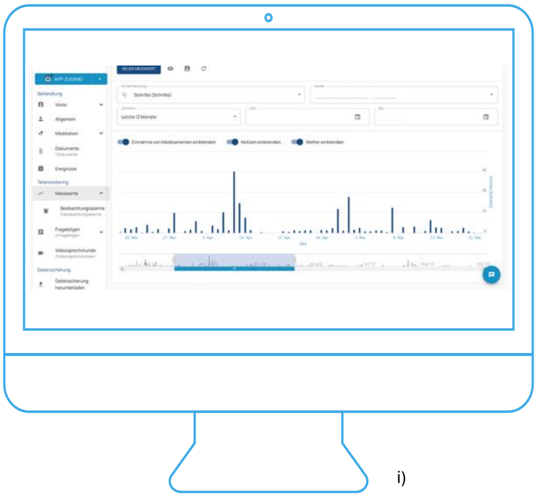

i)

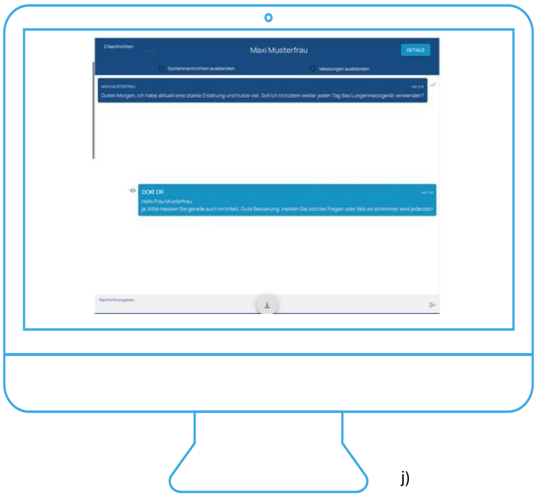

j)
