# Supplementary material for: Feasibility, utility, usability and acceptance of a multimodal telemonitoring for COVID-19 patients in general practitioners practices in Germany: a mixed methods study with patients
Source: BMC Health Serv Res. 2025 Sep 18;25:1203. doi: 10.1186/s12913-025-13455-5 (PMC12447617; doi:10.1186/s12913-025-13455-5)
Supplement: Supplementary file 2 — Supplementary Material 2 [file 12913_2025_13455_MOESM2_ESM.docx]

**Additional File 2**

**Table A2.** COVID-19 initial symptom assessment and health status questionnaire.

| **Initial disease symptoms – Questionnaire Item** | **Prevalent** |
| --- | --- |
| Do you have fever or chills?  Do you have a cough, with or without phlegm?  Do you have difficulty breathing (shortness of breath, breathlessness, wheezing)?  Do you feel lethargic or exhausted?  Do you have concentration difficulties?  Is your sense of smell or taste impaired?  Do you have a sore throat?  Do you have rhinitis?  Do you have a headache or dizziness?  Do you have any pain in your muscles or joints?  Have you any pain in your chest?  Do you have any gastro-intestinal problems (diarrhea, vomiting, nausea)?  Do you have a skin rash?  Do you have any other symptoms? Please explain (Free text)  *Note*. Questionnaire originally in German, translated to English for publication purposes. | yes / no  yes / no  yes / no  yes / no  yes / no  yes / no  yes / no  yes / no  yes / no  yes / no  yes / no  yes / no  yes / no  ____________ |
