# Supplementary material for: Feasibility, utility, usability and acceptance of a multimodal telemonitoring for COVID-19 patients in general practitioners practices in Germany: a mixed methods study with patients
Source: BMC Health Serv Res. 2025 Sep 18;25:1203. doi: 10.1186/s12913-025-13455-5 (PMC12447617; doi:10.1186/s12913-025-13455-5)
Supplement: Supplementary file 3 — Supplementary Material 3 [file 12913_2025_13455_MOESM3_ESM.docx]

**Additional File 3**

**Table A3.** COVID-19 daily symptom questionnaire.

| **Question No. 1** | | | | | | | | | | |
| --- | --- | --- | --- | --- | --- | --- | --- | --- | --- | --- |
| *Do you have shortness of breath today?* | 🞅 | | | | *No* | | | | | |
|  | 🞅 | | | | *I get short of breath with moderate exertion (e.g., when moving around the room)* | | | | | |
|  | 🞅 | | | | *I get short of breath with light exertion (e.g., when washing or dressing)* | | | | | |
|  | 🞅 | | | | *I get short of breath even at rest (e.g., I can hardly speak due to shortness of breath, even when sitting or lying still)* | | | | | |
|  |  | |  | | | |  | |  | |
| **Question No. 2** | | | | | | | | | | |
| *Do you feel tired, exhausted, or sleepy today?* | 🞅 | | | | *No* | | | | | |
|  | 🞅 | | | | *I feel exhausted after moderate exertion (e.g., when moving around the room)* | | | | | |
|  | 🞅 | | | | *I feel exhausted after light exertion (e.g., when washing or dressing)* | | | | | |
|  | 🞅 | | | | *I feel exhausted even at rest (e.g., I can hardly speak due to exhaustion/fatigue, even when sitting or lying still)* | | | | | |
|  |  | |  | | | |  | |  | |
| **Question No. 3** | | | | | | | | | | |
| *Do you have chills today?* | 🞅 | | | | *No* | | | | | |
|  | 🞅 | | | | *I have mild chills* | | | | | |
|  | 🞅 | | | | *I have moderate chills* | | | | | |
|  | 🞅 | | | | *I have recurrent or persistent severe chills* | | | | | |
|  |  | |  | | | |  | |  | |
| **Question No. 4** | | | | | | | | | | |
| *Do you find it difficult to concentrate today?* | | 🞅 | | | | *No* | | | | |
|  |  | 🞅 | | | | *I have slight (occasional) difficulty concentrating* | | | | |
|  |  | 🞅 | | | | *I have moderate difficulty concentrating, which reduces my ability to read or have a conversation* | | | | |
|  |  | 🞅 | | | | *I have great difficulty concentrating. I cannot read or have a conversation without difficulty* | | | | |
|  | |  | |  | | | |  | |  |
| **Question No. 5** | | | | | | | | | | |
| *Do you have muscle pain or joint pain today?* | | 🞅 | | | | *No* | | | | |
|  |  | 🞅 | | | | *I have mild muscle or joint pain* | | | | |
|  |  | 🞅 | | | | *I have moderate muscle or joint pain* | | | | |
|  |  | 🞅 | | | | *I have severe muscle or joint pain* | | | | |

| **Question No. 6** | | | | | |
| --- | --- | --- | --- | --- | --- |
| *Do you have any*  *other additional symptoms?* | 🞅 | | *No* | | |
|  | 🞅 | | *Yes* | | |
|  |  |  | |  |  |
| **Question No. 7** | | | | | |
| *What other symptoms do you have? (If there are no other complaints, please leave blank)* | Free text answer | |  | | |

*Note.* Questionnaire originally in German, translated to English for publication purposes.
