# Supplementary material for: Feasibility, utility, usability and acceptance of a multimodal telemonitoring for COVID-19 patients in general practitioners practices in Germany: a mixed methods study with patients
Source: BMC Health Serv Res. 2025 Sep 18;25:1203. doi: 10.1186/s12913-025-13455-5 (PMC12447617; doi:10.1186/s12913-025-13455-5)
Supplement: Supplementary file 4 — Supplementary Material 4 [file 12913_2025_13455_MOESM4_ESM.docx]

**Additional File 4**

**Table A4.** In-house questionnaire: Evaluation of app and measuring devices.

| **Questionnaire Item** |
| --- |
| **I cope well with the app and measuring devices** |
| Completely agree |
| Partially agree |
| Partially disagree |
| Completely disagree |
| **I could easily integrate the measuring process into everyday life** |
| Partially agree |
| Partially disagree |
| Completely disagree |
| **I experienced uncertainty when using the app and the measuring devices** |
| Completely agree |
| Partially agree |
| Partially disagree |
| Completely disagree |
| **My health was severely impaired due to COVID-19** |
| Completely agree |
| Partially agree |
| Partially disagree |
| Completely disagree |
| **I had the feeling that my COVID-19 treatment benefited from telemonitoring** |
| Completely agree |
| Partially agree |
| Partially disagree |
| Completely disagree |
| **I received help in using the app and measuring devices** |
| Regularly |
| Occasionally |
| Rarely |
| Never |
| **I previously kept records of my health (e.g., blood pressure or blood sugar measurements, headache diary)** |
| Regularly |
| Occasionally |
| Rarely |
| Never |
| **Overall rating of the telemonitoring** |
| Very good |
| Rather good |
| Rather poor |
| Very poor |
| **I would recommend the app and measuring devices to friends and family** |
| Yes |
| Yes, probably |
| No, probably not |
| No |

*Note.* Questionnaire originally in German, translated to English for publication purposes.
