# Supplementary material for: Feasibility, utility, usability and acceptance of a multimodal telemonitoring for COVID-19 patients in general practitioners practices in Germany: a mixed methods study with patients
Source: BMC Health Serv Res. 2025 Sep 18;25:1203. doi: 10.1186/s12913-025-13455-5 (PMC12447617; doi:10.1186/s12913-025-13455-5)
Supplement: Supplementary file 5 — Supplementary Material 5 [file 12913_2025_13455_MOESM5_ESM.docx]

**Additional File 5**

**Table A5** Interview guide for the semi-structured interviews.

|  | **Question** | **Objective** |
| --- | --- | --- |
| **Experience with telemonitoring (TAM)** | **“How did you get along with the app and devices in the Covid19@home study?"** (Could you tell us a bit more about your experiences with telemonitoring)? | Technology Acceptance Model (TAM) Ease of Use |
|  | **“How did you carry out the measurements on a normal day?"** (Did you perform the measurements yourself or did someone help you? At what times of the day do you generally take the measurements?) | Measurement management |
|  | **"How did you experience the measurement process?"**  (Did the devices work reliably? Did you perform the measurements regularly? If not, why not? Were all the measurements transmitted to the doctor? Was it easy to take the measurements?) | TAM Perceived Usefulness  TAM Ease of Use  TAM Intention to Use |
|  | **"Did you ever receive feedback from the app that your values were in a critical range?"**  (How did you deal with that? What did you do when it happened? Was there anyone you could report to when something like that occurred?) | Experience with critical measurements |
|  | **"Looking back over the last few weeks, did you feel comfortable or uncomfortable using the app overall?** | TAM Attitude toward Technology |
|  | **"Did you have any previous experience of using health apps for telemonitoring?"** (If so, what apps did you use?) | TAM Habits |
| **Study setting** | **“Now I would like to talk about the Covid19@home project.**  **How did you come to participate?"** (What motivated you to participate?) | Motivation |
|  | **"What were your expectations when you agreed to participate?"** (What did you think would change for you? What did you think would change for your GP?) | Expectations |
|  | **"How did communication with your GP work?"** (Did it depend on monitoring data whether you contacted your GP? Did it depend on monitoring data whether your GP contacted you? Did your GP initiate anything else, e.g., a home visit, hospitalization?) | Communication with GP |
| **Reflection** | **"Looking back, what do you think is good about telemonitoring overall?"** | Enabling Factors |
|  | **"What bothers you about telemonitoring?”** (What disadvantages do you see for yourself personally or for other patients in using telemonitoring? From your point of view, why might patients have dropped out of the study or not wished to participate? Did you ever consider dropping out of the study?) | Hindering factors |
|  | **“Would you recommend using the app and devices to your family and friends?"** | Recommendation |
| **Suggestions** | **“Do you have any specific suggestions for improvement?” (**Regarding the application itself? Regarding communication with your GP? Regarding support by the study team?) | Improvement |
| **Conclusion** | **Is there anything else you would like to tell us? Do you have any further questions, wishes or comments?** | Closing |

*Note*. Interview guide originally in German, translated to English for publication purposes. TAM=Technology Acceptance Model according to Gagnon et al. (2012)
